# Exploiting the Massey Gap

**DOI:** 10.3390/e22121398

**Published:** 2020-12-11

**Authors:** Andrei Tănăsescu, Pantelimon George Popescu

**Affiliations:** Department of Computer Science and Engineering, University Politehnica of Bucharest, Splaiul Independentei 313 (6), 060042 Bucharest, Romania; andrei.tanasescu@mail.ru

**Keywords:** shannon entropy, guessing, massey inequality

## Abstract

In this paper, we find new refinements to the Massey inequality, which relates the Shannon and guessing entropies, introducing a new concept: the Massey gap. By shrinking the Massey gap, we improve all previous work without introducing any new parameters, providing closed-form strict refinements, as well as a numerical procedure improving them even further.

## 1. Introduction

The guessing entropy associated with a probabilistic vector $p=\left(p_{1},\;p_{2},\;\cdots ,\;p_{n}\right)$ with $p_{1}\geq \cdots \geq p_{n}>0$ is the expected value of the random variable $G\left(p\right)$ given by $\mathrm{Pr}\left[G\left(p\right)=i\right]=p_{i}\forall i\in \bar{1,n}$, i.e., $E\left[G\left(p\right)\right]=\sum_{i=1}^{n}ip_{i}$, and it corresponds to the optimal average number of binary questions required guessing the value of a random variable distributed according to p. Meanwhile, the Shannon entropy of p is $H\left(p\right)=-\sum_{i=1}^{n}p_{i}\log p_{i}$, and the first relation between them was provided by J. Massey in [1], namely that if $H\left(p\right)\geq 2$, then $E\left[G\left(p\right)\right]\geq 2^{H\left(p\right)-2}+1$.

While the Massey inequality has been shown to have many applications such as password guessing [2] and encourages multiple developments such as moments inequalities (e.g., [3,4]), there are only two known direct refinements to the Massey inequality, both of which were published in 2019. In an ISITpaper, Popescu and Choudary [5] found that:

(1) $$E\left[G\left(p\right)\right]\;\geq \;2^{H\left(p\right)+2p_{n}-2}+1-p_{n}$$

(2) $$\geq 2^{H\left(p\right)+p_{n}-2}+1-\frac{1}{2}p_{n}$$

(3) $$\geq 2^{H\left(p\right)-2}+1,$$

subject to the same conditions as the Massey inequality. Meanwhile, Rioul’s inequality [6], published in a CHESpaper [7], states that for all values of $H\left(p\right)$, we have:

(4) $$E\left[G\left(p\right)\right]>2^{H\left(p\right)}/e$$

a bound that is similar to the Massey inequality and refines it when $H\left(p\right)\geq \log\frac{e}{1-e/4}$.

Remarkably, the refinement in [5] is actually strict since p has finite support, $\left|p\right|=n\leq 1/\inf p<\infty$. In fact, applying the method from [5], it can be easily observed that Equation (4) could be further improved. For example, applying just the first step of the method presented in [5], we can obtain that for $H\left(p\right)\geq \log\frac{e}{2\ln2}$, we have a new further refinement of Equation (4),

$$E\left[G\left(p\right)\right]>2^{H\left(p\right)+p_{n}}/e-\frac{1}{2}p_{n}>2^{H\left(p\right)}/e.$$

Motivated by this insight, in this paper, we find and fully investigate a method to refine an inequality regarding finite support vectors using only itself. Here, we apply our analysis to the famous Massey inequality and explore multiple avenues of applying our method: shrinking the Massey gap.

## 2. Results

In this section, we present our results, as well as their proofs. In Section 2.1, we find a refinement over the initial result of [5], i.e., Equation (2), which we improve in Section 2.2 beyond even the strongest result of [5], i.e., Equation (1). Then, in Section 2.3, we introduce a new concept, the Massey gap, and use it to generalize the method in Section 2.2. Next, in Section 2.4, we show how using the Massey gap, we can almost always improve our refinement, by rebalancing the middle of the distribution, and finally, in Section 2.5, we find closed-form easy to compute refinements over the work of [5].

### 2.1. An Improved Refinement to the Massey Inequality

In this section, we find a new relation between the Shannon and guessing entropy, dependent on the minimal probability of a given distribution, refining the initial bound, Equation (2), from the work of [5].

Consider a decreasing probabilistic vector $p\;=\;\left(p_{1},\;p_{2},\;\cdots ,\;p_{n}\right)$ with $H\left(p\right)\geq 2$. Inspired by the trick in [5], we construct the new probabilistic vector $q\;=\;\left(p_{1},\;p_{2},\;\cdots ,\;p_{n-1},\;\left(1-\alpha \right)p_{n},\;\alpha p_{n}\right)$, which is decreasing and strictly positive if and only if $\alpha \in \left(0,\;1/2\right]$. From the grouping property of entropy,

(5) $$H\left(q\right)\;=\;H\left(p\right)\;+\;p_{n}h\left(\alpha \right),$$

where $h\left(\alpha \right)=-\alpha \log\alpha -\left(1-\alpha \right)\log\left(1-\alpha \right)$ is the binary entropy function. Moreover, we calculate:

(6) $$E\left[G\left(q\right)\right]\;=\;E\left[G\left(p\right)\right]\;+\;\alpha p_{n}.$$

Thus, because $H\left(q\right)\geq 2$, we can apply the Massey inequality for q to obtain:

$$\begin{array}{c} E\left[G\left(p\right)\right]=E\left[G\left(q\right)\right]-\alpha p_{n}\geq 2^{H\left(q\right)-2}+1-\alpha p_{n}=2^{H\left(p\right)+p_{n}h\left(\alpha \right)-2}+1-\alpha p_{n}. \end{array}$$

Now, we note that $h\left(0\right)=0$ and $h\left(1/2\right)=1>\frac{1}{2}\frac{1}{\ln2}\approx 0.7213$; thus, by the strict concavity of *h*, we have $h\left(\alpha \right)>\alpha \frac{1}{\ln2}$. Using the inequality $2^{x}>1+x\ln2$ for $x=p_{n}h\left(\alpha \right)>0$, we find that:

$$\begin{array}{cc} 0 & \leq p_{n}\left(h\left(\alpha \right)\ln\;2-\alpha \right)<2^{p_{n}h\left(\alpha \right)}-1-\alpha p_{n}\\ & \leq 2^{H\left(p\right)-2}\left(2^{p_{n}h\left(\alpha \right)}-1\right)-\alpha p_{n}=\left(2^{H\left(p\right)+p_{n}h\left(\alpha \right)-2}+1-\alpha p_{n}\right)-\left(2^{H\left(p\right)-2}+1\right), \end{array}$$

i.e., our lower bound is always strictly tighter than Massey’s,

(7) $$E\left[G\left(p\right)\right]\geq 2^{H\left(p\right)+p_{n}h\left(\alpha \right)-2}+1-\alpha p_{n}>2^{H\left(p\right)-2}+1\;\forall \alpha \in \left(0,1/2\right].$$

For the particular value α=1/2, the bound above produces the initial bound, Equation (2), of [5]. Thus, we conclude this subsection with the following refinement to Equation (2) summarizing the above:

**Theorem** **1.** *For strictly positive descending probabilistic vectors $p\in R^{n}$ with $H\left(p\right)\geq 2$, we have:*

$$\begin{array}{cc} E\left[G\left(p\right)\right] & \geq \underset{\alpha \in \left[0,1/2\right]}{\sup}2^{H\left(p\right)+p_{n}h\left(\alpha \right)-2}+1-\alpha p_{n}\geq 2^{H\left(p\right)+p_{n}-2}+1-\frac{1}{2}p_{n}\\ & >\underset{\alpha \in \left[0,1/2\right]}{\inf}2^{H\left(p\right)+p_{n}h\left(\alpha \right)-2}+1-\alpha p_{n}=2^{H\left(p\right)-2}+1. \end{array}$$

**Proof.**  The first inequality follows taking the supremum over α in Equation (7). The second and third inequality follow taking α=1/2, which yields the right-hand side in Equation (2), where the strictness of the last inequality follows from the strictness of (7), the right-hand side of which is the bound obtained taking α=0. □

Up to this point, we found a direct refinement over the initial result of [5]. In the next section, we will leverage Theorem 1 in order to refine the strongest result, the bound in Equation (1).

### 2.2. Refinement through Generalization

In this section, we extend the reasoning of [5] to improve our bound from Theorem 1 beyond (1). We leverage a technique previously used in [8], namely applying the Massey inequality to increasingly complicated perturbations of the input in order to obtain ever tighter bounds for the original input.

Given an initial decreasing probabilistic vector p, we construct a list of probabilistic sequences $\left\{Q_{k}\right\}$, which we recursively define according to the procedure in the previous subsection.

We begin by fixing an arbitrary parameter $\alpha \in \left[0,1/2\right]$ as in the section above. Now, we explicate our construction. Denoting by $Q_{k,i}$ the *i*th component of the sequence $Q_{k}$, we define the terms of the list $\left\{Q_{k}\right\}$ as follows. We let the support of the first term coincide with p, i.e., $Q_{0}=\left(p_{0},\;p_{1},\;\cdots ,\;p_{n},\;0,\;0,\;\cdots ,\;0,\;\cdots \right)$, and we define the other terms by recurrence:

$$Q_{k+1}=\;\left(Q_{k,0},\;Q_{k,1},\;\cdots ,\;Q_{k,n+k-1},\;\left(1-\alpha \right)Q_{k,n+k},\;\alpha Q_{k,n+k},\;0,\;0,\;\cdots ,\;0,\;\cdots \right).$$

Now, we expand the recursion relation for the Shannon entropy, $H\left(Q_{k+1}\right)$, and guessing entropy $E\left[G\left(Q_{k+1}\right)\right]$, which are analogous to Equations (5) and (6), respectively, to obtain:

$$\begin{array}{cc} H\left(Q_{k+1}\right) & =H\left(Q_{k}\right)+Q_{k,n+k}h\left(\alpha \right)=H\left(Q_{k}\right)+\alpha^{k}p_{n}h\left(\alpha \right)\\ & =H\left(p\right)+p_{n}h\left(\alpha \right)\sum_{j=0}^{k}\alpha^{j}=H\left(p\right)+p_{n}h\left(\alpha \right)\frac{1-\alpha^{k+1}}{1-\alpha },\\ E\left[G\left(Q_{k+1}\right)\right] & =E\left[G\left(Q_{k}\right)\right]+\alpha Q_{k,n+k}=E\left[G\left(Q_{k}\right)\right]+\alpha^{k+1}p_{n}\\ & =E\left[G\left(p\right)\right]+p_{n}\sum_{j=0}^{k}\alpha^{j+1}=E\left[G\left(p\right)\right]+p_{n}\alpha \frac{1-\alpha^{k+1}}{1-\alpha }, \end{array}$$

so, at each step *k*, we can use $Q_{k+1}$ to obtain an approximation of $E\left[G\left(Q_{k}\right)\right]$ that is tighter than Massey’s. This procedure allows us to obtain ever tighter approximations of the guessing entropy.

For example, fixing an arbitrary mixing rate α>0 and step number k>0, we apply the Massey inequality for the distribution $Q_{k+1}$ and find that as per Equation (7),

$$\begin{array}{c} E\left[G\left(Q_{k}\right)\right]=E\left[G\left(Q_{k+1}\right)\right]-\alpha Q_{k,n+k}\geq 2^{H\left(Q_{k+1}\right)-2}+1-\alpha Q_{k,n+k}>2^{H\left(Q_{k}\right)-2}+1. \end{array}$$

Now, this refinement trickles down producing ever tighter bounds on the guessing entropy of lower index elements of the list up to the initial element $p=Q_{0}$,

$$\begin{array}{cc} E\left[G\left(p\right)\right] & =E\left[G\left(Q_{k}\right)\right]-p_{n}\alpha \frac{1-\alpha^{k}}{1-\alpha }=E\left[G\left(Q_{k}\right)\right]+\sum_{j=0}^{k-1}\left(E\left[G\left(Q_{j}\right)\right]-E\left[G\left(Q_{j+1}\right)\right]\right)\\ & \geq 2^{H\left(Q_{k}\right)-2}+1+\sum_{j=0}^{k-1}\left(E\left[G\left(Q_{j}\right)\right]-E\left[G\left(Q_{j+1}\right)\right]\right)\\ & >2^{H\left(Q_{k-1}\right)-2}+1+\sum_{j=0}^{k-2}\left(E\left[G\left(Q_{j}\right)\right]-E\left[G\left(Q_{j+1}\right)\right]\right)\\ & >\cdots >2^{H\left(Q_{0}\right)-2}+1=2^{H\left(p\right)-2}+1, \end{array}$$

where the tightest of the enumerated bounds is:

$$\begin{array}{cc} E\left[G\left(p\right)\right] & \geq 2^{H\left(Q_{k}\right)-2}+1+\sum_{j=0}^{k-1}\left(E\left[G\left(Q_{j}\right)\right]-E\left[G\left(Q_{j+1}\right)\right]\right)\\ & =2^{H\left(p\right)+p_{n}h\left(\alpha \right)\frac{1-\alpha^{k}}{1-\alpha }-2}+1-p_{n}\alpha \frac{1-\alpha^{k}}{1-\alpha }, \end{array}$$

which as we have shown increases with *k* up to the limit:

(8) $$\begin{array}{c} E\left[G\left(p\right)\right]\geq 2^{H\left(p\right)+p_{n}\frac{h\left(\alpha \right)}{1-\alpha }-2}+1-p_{n}\frac{\alpha }{1-\alpha }\;\forall \alpha \in \left[0,1/2\right], \end{array}$$

where the optimal value of α will be discussed in the following sections.

We now conclude this section with an even stronger refinement of the Massey inequality.

**Theorem** **2.** *For strictly positive descending probabilistic vectors $p\in R^{n}$ with $H\left(p\right)\geq 2$, we have:*

$$\begin{array}{cc} E\left[G\left(p\right)\right] & \geq \underset{\alpha \in \left[0,1/2\right]}{\sup}2^{H\left(p\right)+\frac{h\left(\alpha \right)}{1-\alpha }p_{n}-2}+1-\frac{\alpha }{1-\alpha }p_{n}\\ & \geq 2^{H\left(p\right)+2p_{n}-2}+1-p_{n}>2^{H\left(p\right)-2}+1. \end{array}$$

**Proof.**  The first inequality follows taking the supremum over α in Equation (8) and the rest taking α=1/2. □

Furthermore, note that Theorem 2 is a refinement over the strongest result of [5], namely Equation (1).

### 2.3. The Massey Gap

In this section, we reevaluate the scaffolding we used to prove Theorem 2 and re-frame our previous technique as an optimization process applied to an objective function we name the Massey gap, the function $M\left(x\right)$ described below.

The first observation that we make is that Theorem 2 also applies to descending probabilistic infinite sequences p, by taking *n* to be the size of its support, $\left|p\right|$. Moreover, we notice that the core of the argument was not the precise construction of the list $\left\{Q_{k}\right\}$, but rather the fact that:

$$2^{H\left(Q_{k}\right)-2}+1+E\left[G\left(Q_{k-1}\right)\right]-E\left[G\left(Q_{k}\right)\right]>2^{H\left(Q_{k-1}\right)-2}+1\;\forall k\geq 1,$$

i.e., the sequences $\left\{Q_{k}\right\}$ incrementally optimize the objective function $M\left(x\right)=E\left[G\left(x\right)\right]-2^{H\left(x\right)-2}-1$, with tightness being achieved if and only if $\lim_{k\to \infty }M\left(Q_{k}\right)=0$.

Motivated by this insight, we now take an alternative view and focus not on the precise sequence of distributions, but rather the objective function $M\left(x\right)$, which we name the Massey gap.

This view leads us to the following useful result:

**Remark** **1.** *Let $\left\{X_{k}\right\}$ be a list of probabilistic sequences with $H\left(X_{k}\right)\geq 2$ and $M\left(X_{k}\right)\geq M\left(X_{k+1}\right)\geq 0$ for all values of k. Then, the sequence $y_{k}=2^{H\left(X_{k}\right)-2}+1+E\left[G\left(X_{0}\right)\right]-E\left[G\left(X_{k}\right)\right]$ is increasing.*

Notice that this remark provides a sequence $\left\{y_{k}\right\}$ of ever tighter lower bounds on $E\left[G\left(X_{0}\right)\right]$, the weakest of which, $y_{0}$, reduces to the Massey inequality, while the strongest is given by $\lim_{k\to \infty }y_{k}$. As an example, considering the list $X_{k}:=Q_{k}$, this limit coincides with the result of Theorem 2.

### 2.4. Mixing Probabilities

In the previous section, we presented a refinement to (1) by generalizing the tail rebalancing method of [5]. In this section, we investigate whether we can obtain even better results by applying the same rebalancing technique in the middle of the distribution, rather than at the tail.

In the previous section, we showed how Theorem 2 follows from Remark 1 for some particular $\left\{X_{k}\right\}$. However, this option is not necessarily unique. In this section, we consider alternative methods of optimizing the Massey gap, that is we fix an arbitrary intermediary configuration $X_{k}$, and we find conditions on the existence of available $X_{k+1}$ that decrease the Massey gap, i.e., $M\left(X_{k}\right)\geq M\left(X_{k+1}\right)$.

In order to simplify the notation, for the remainder of this section, we denote the available configuration $X_{k}$ by p and the potential next configuration $X_{k+1}$ by q.

Now, we are ready for a trick similar to the one in Section 2.2: we fix an arbitrary integer i∈N such that $p_{i}\neq 0$ and an arbitrary mixing rate $\alpha \in \left(0,1/2\right]$ and consider q of the form:

(9) $$q=\left(p_{0},\;p_{1},\;\cdots ,\;p_{i-1},\;\left(1-\alpha \right)p_{i}+\alpha p_{i+1},\;\alpha p_{i}+\left(1-\alpha \right)p_{i+1},\;p_{i+2},\;p_{i+3},\;\cdots \right),$$

with the same support as p.

For q as defined above in Equation (9), we find that $E\left[G\left(q\right)\right]=E\left[G\left(p\right)\right]+\alpha \left(p_{i}-p_{i+1}\right)$ while:

$$H\left(q\right)=H\left(p\right)+\left(p_{i}+p_{i+1}\right)\left(h\left(\frac{q_{i}}{q_{i}+q_{i+1}}\right)-h\left(\frac{p_{i}}{p_{i}+p_{i+1}}\right)\right);$$

thus, the condition $M\left(p\right)\geq M\left(q\right)$ is equivalent to:

(10) $$h\left(\frac{q_{i}}{q_{i}+q_{i+1}}\right)\geq h\left(\frac{p_{i}}{p_{i}+p_{i+1}}\right)+\frac{1}{p_{i}+p_{i+1}}\log\left(1+\frac{\alpha \left(p_{i}-p_{i+1}\right)}{2^{H\left(p\right)-2}}\right),$$

which, as we have seen in the previous section, always holds at least for some *i*, namely $i=\left|p\right|$.

Now, we check whether alternative values for *i* may be eligible, i.e., whether we may mix probabilities in the middle of the distribution. Using the inequality (Tighter bounds may also be used.) $1-h\left(\frac{1+y}{2}\right)\leq \frac{y^{2}}{2\ln2}$ with $y=\frac{q_{i}-q_{i+1}}{q_{i}+q_{i+1}}=\left(1-2\alpha \right)\frac{p_{i}-p_{i+1}}{p_{i}+p_{i+1}}$, we find that:

$$h\left(\frac{q_{i}}{q_{i}+q_{i+1}}\right)\geq 1-{\left(1-2\alpha \right)}^{2}{\left(\frac{p_{i}-p_{i+1}}{p_{i}+p_{i+1}}\right)}^{2}$$

Thus, to satisfy $M\left(p\right)\geq M\left(q\right)$, corresponding to a decrease in the Massey gap, it is sufficient that:

$$\begin{array}{cc} 1-{\left(1-2\alpha \right)}^{2}{\left(\frac{p_{i}-p_{i+1}}{p_{i}+p_{i+1}}\right)}^{2}\geq & h\left(\frac{p_{i}}{p_{i}+p_{i+1}}\right)+\frac{1}{p_{i}+p_{i+1}}\log\left(1+\frac{\left(p_{i}-p_{i+1}\right)}{2^{H\left(p\right)-2}}\right). \end{array}$$

i.e.,

(11) $$\alpha \geq \frac{1}{2}-\frac{1}{2}\frac{p_{i}+p_{i+1}}{p_{i}-p_{i+1}}{\left[1-h\left(\frac{p_{i}}{p_{i}+p_{i+1}}\right)-\frac{1}{p_{i}+p_{i+1}}\log\left(1+\frac{\left(p_{i}-p_{i+1}\right)}{2^{H\left(p\right)-2}}\right)\right]}^{1/2}\geq 0,$$

so depending on the value of $H\left(p\right)$, multiple indices *i* and mixing ratios α may be selected for rebalancing, as outlined in the following

**Theorem** **3.** *Let p be a descending probabilistic sequence with $H\left(p\right)\geq 2$ and i∈N be such that $p_{i}\neq 0$ and $\alpha \in \left(0,1/2\right]$ satisfy Equation (10) or, sufficiently, Equation (11). Then, defining q as in Equation (9), we have:*

$$E\left[G\left(p\right)\right]\geq 2^{H\left(q\right)-2}+1-\alpha \left(p_{i}-p_{i+1}\right)\geq 2^{H\left(p\right)-2}+1.$$

**Proof.**  We have shown that Equation (11) is a sufficient condition for us to have $M\left(p\right)\geq M\left(q\right)$; thus, applying Remark 1 to the sequence $X=\left(p,\;q,\;q,\;q,\;\cdots \right)$, the result follows. □

We conclude this section by remarking that Theorem 3 has the potential to further refine bounds obtained using Theorem 1 depending on the form of the distribution p.

### 2.5. Strict Refinement and Optimal Mixing Rate

In this section, we consider the problem of finding the optimal mixing rate α.

We notice that α must be a critical point of the difference between the sides of (10), which is:

$$f\left(\alpha \right)=\frac{p_{i}-p_{i+1}}{p_{i}+p_{i+1}}\left[\log\frac{q_{i}}{q_{i+1}}-\frac{1}{\alpha \left(p_{i}-p_{i+1}\right)+2^{H\left(p\right)-2}}\right].$$

For example, we notice that if $H\left(p\right)\geq 2$, then the right denominator is greater than one and:

$$f\left(1/2\right)=-\frac{p_{i}-p_{i+1}}{p_{i}+p_{i+1}}\frac{1}{\alpha \left(p_{i}-p_{i+1}\right)+2^{H\left(p\right)-2}}<0$$

so the mixing rate α=1/2 is never optimal; thus, Theorem 2 is a strict refinement over the work of [5].

Investigating whether q actually decreases the Massey gap, we now consider the optimality of α=0. In this case, $q_{i}=p_{i}$ and $q_{i+1}=p_{i+1}$; thus, when $p_{i}\geq p_{i+1}2^{2^{2-H\left(p\right)}}$, then $f\left(0\right)>0$, so α=0 is certainly sub-optimal as $M\left(q\right)-M\left(p\right)$ is increasing around zero.

Moreover, we note that the simple upper bound:

$$f\left(\alpha \right)\leq \frac{p_{i}-p_{i+1}}{p_{i}+p_{i+1}}\left[\log\frac{q_{i}}{q_{i+1}}-\frac{1}{\left(p_{i}-p_{i+1}\right)+2^{H\left(p\right)-2}}\right]$$

imposes that the optimal mixing ratio $\alpha^{*}$ satisfy:

$$\frac{p_{i+1}+p_{i}}{q_{i+1}}-1=\frac{q_{i}}{q_{i+1}}\geq 2^{{\left(\left(p_{i}-p_{i+1}\right)+2^{H\left(p\right)-2}\right)}^{-1}}$$

i.e.,

$$q_{i+1}\leq \frac{p_{i}+p_{i+1}}{1+2^{{\left(\left(p_{i}-p_{i+1}\right)+2^{H\left(p\right)-2}\right)}^{-1}}}$$

and hence:

(12) $$\begin{array}{cc} \alpha^{*}\leq & \frac{1}{p_{i}-p_{i+1}}\left[-p_{i+1}+\frac{p_{i}+p_{i+1}}{1+2^{{\left(\left(p_{i}-p_{i+1}\right)+2^{H\left(p\right)-2}\right)}^{-1}}}\right]\leq \frac{1}{2}, \end{array}$$

which is a non-trivial bound.

For example, to apply Equation (12) to the particular case of mixing only at the tail of the distribution, i.e., Theorem 2, we take i=n, hence $p_{i+1}=0$, and substituting in Equation (12), we obtain the requirement:

$$\alpha^{*}\leq 1/\left(1+2^{1/\left(p_{n}+2^{H\left(p\right)-2}\right)}\right)<1/2$$

on the mixing rate realizing the supremum, so we have a closed-form strict refinement over Equation (2):

**Theorem** **4.** *Let p be a descending probabilistic sequence with $H\left(p\right)\geq 2$ and i∈N be such that $p_{i}>2^{2^{2-H\left(p\right)}}p_{i+1}$, and let $\alpha =1/\left(1+2^{1/\left(p_{n}+2^{H\left(p\right)-2}\right)}\right)$. Then, defining q as in Equation (9), we have:*

$$E\left[G\left(p\right)\right]\geq 2^{H\left(q\right)-2}+1-\alpha \left(p_{i}-p_{i+1}\right)>2^{H\left(p\right)+p_{\left|p\right|}-2}+1-\frac{1}{2}p_{\left|p\right|}\geq 2^{H\left(p\right)-2}+1.$$

We end this section by remarking that Theorem 4 can be used in conjunction with Theorem 2, complementing the construction of the list of sequences defined in the proof of Theorem 2.

## 3. Discussion

In this section, we discuss in a more detailed manner the context of our work, evaluate the saturation conditions of the discussed bounds, and present prospects for future work.

We begin our discussion by taking a closer look at the most prevalently used form of the Massey inequality, namely Equation (3). Notably, this inequality only holds for the case that $H\left(p\right)\geq 2$, suggesting that its nice shape is a case of form vs. function, as illustrated below.

Massey [1] found that the set of probabilistic vectors of arbitrary length, which have a fixed guessing entropy *A*, are convex for any given *A*; thus, by strict convexity, the Shannon entropy attains its maximum over this set at an interior point. Using Lagrange multipliers, Massey found that the critical p must be a geometric distribution, and imposing the condition $E\left[G\left(p\right)\right]=A$, he found $p_{i}=\frac{1}{A-1}{\left(1-\frac{1}{A}\right)}^{i}$, hence:

(13) $$H\left(p\right)=\log\left(A-1\right)+\log\left[{\left(1-\frac{1}{A}\right)}^{-A}\right],$$

Because this distribution maximizes the Shannon entropy, for arbitrary distributions p, we have:

(14) $$H\left(p\right)\leq \log\left(E\left[G\left(p\right)\right]-1\right)+\log\left[{\left(1-\frac{1}{E\left[G\left(p\right)\right]}\right)}^{-E\left[G\left(p\right)\right]}\right],$$

an unwieldy form that is saturated only for geometric distributions. Thus, the bound in Equation (14) is only ever saturated when $\left|p\right|=\infty$; therefore, the Massey inequality is never tight for distributions encountered in most applications such as cryptography.

To find a simpler form of Equation (14), Massey noticed that when A≥2, the second term of Equation (13) is at most two, with equality if and only if A=2. Thus, when $H\left(p\right)\geq 2$, $E\left[G\left(p\right)\right]\geq 2^{H\left(p\right)-2}+1$. However, the simplicity of this form impacts its tightness, as we can remark:

**Remark** **2.** *While the well-known form of Massey’s lower bound Equation (3) is only saturated when $p_{i}=1/2^{i}$, his original inequality Equation (14) is saturated by all geometric distributions, i.e., for all values of $E\left[G\left(p\right)\right]$.*

From this remark, we conclude that any inequality derived from an application of the Massey inequality is saturated if and only if the distribution for which the Massey inequality is ultimately applied is the geometric distribution of ratio 1/2. It follows that for the initial result of [5], Equation (2), as well as for our own initial result from Theorem 1, the bounds are saturated only for the geometric distribution of the ratio 1/2, even if they are tighter than the Massey inequality.

However, when those inequalities are applied after an unbounded number of steps, it is possible to obtain bounds saturated by more inputs. Indeed, for the stronger result of [5], as well as our own stronger result from Theorem 2, the bounds are saturated for any truncation of the geometric distribution of the ratio 1/2, i.e., when the original distribution has the form $p=\left(1/2,\;1/4,\;\cdots ,\;1/2^{n-2},\;1/2^{n-1},\;1/2^{n-1}\right)$ for some n≥2, i.e., for a countable range of $E\left[G\left(p\right)\right]$.

It is then natural to ask how the tightness improvement in Theorems 1 and 2 is reflected in saturation conditions. To this end, we notice that the limiting factor is the requirement that the limit of the list of probabilistic distributions is required to be the geometric distribution of the ratio 1/2; hence, for saturation, we necessarily have α=1/2, i.e., this is a direct consequence of using the nice form of the Massey inequality, Equation (3). As such, we propose the open question of finding a similar technique exploiting the Massey gap with respect to the original form of the Massey inequality, Equation (14).

We continue our discussion by comparing the many refinements to the Massey inequality that we have presented on a simple numerical example. Let $p=\left(p_{1},\;\cdots ,\;p_{10}\right)$ be the descending probabilistic vector containing the first nine terms of the probabilistic geometric sequence of the ratio 0.8,

$$p=\left(\begin{array}{cccccccccc} \frac{1}{5} & \frac{4}{25} & \frac{262144}{1953125} & \frac{16}{125} & \frac{64}{625} & \frac{256}{3125} & \frac{1024}{15625} & \frac{4096}{78125} & \frac{16384}{390625} & \frac{65536}{1935125} \end{array}\right),$$

with Shannon entropy $H\left(p\right)\approx 3.32516>\log\frac{e}{1-e/4}>2$ and guessing entropy $E\left[G\left(p\right)\right]\approx 4.02939$. The state-of-the-art provides various lower bounds on the guessing entropy: 3.18126 from the Massey inequality, 3.21581 from the weaker result Equation (2) of [5], 3.25157 from their stronger result Equation (1), and 3.20977 from Rioul’s inequality [6,7]. Our new lower bounds for this example are: 3.21767 as the supremum in Theorem 1 taking α≈0.38977 (improving Equation (2)), 3.25157 as the supremum in Theorem 2 taking α=0.5 (matching Equation (1)), and 3.21751 as the explicit value in Theorem 4 taking i=10 (improving Equation (2)). In this particular case, Theorem 3 cannot be applied for i≠10 as $p_{i}/p_{i+1}\leq 1.2$, while $2^{2^{2-H\left(p\right)}}\approx 1.3189$. We notice that for this example, Theorem 2 matches the stronger result of [5].

The final topic of our discussion addresses a direction that was previously left unaddressed in the literature, rebalancing the initial distribution. We take an example and use it to showcase the effectiveness of the approach in Theorem 3. Considering the initial distribution $p=\left(5/8,\;1/8,\;1/8,\;1/2^{16},\;1/2^{16},\;\cdots ,\;1/2^{16}\right)$, we notice that $H\left(p\right)\geq 1/8\log\;2^{16}=2$, and moreover, $p_{1}/p_{2}=5\geq 2^{2^{2-H\left(p\right)}}$. Hence, according to Section 2.4, we may rebalance our distribution, even at its head. Explicitly taking i=1 and $\alpha =1/5<1/\left(1+2^{1/\left(2^{-16}+2^{H\left(p\right)-2}\right)}\right)$, we construct $q=\left(21/40,\;19/40,\;1/8,\;1/2^{16},\;1/2^{16},\;\cdots ,\;1/2^{16}\right)$ such that $M\left(p\right)\geq M\left(q\right)\geq 0$. From Remark 1, it follows that each such rebalancing can be prepended to the list construction in Section 2.2.

However, continuing our example, we note that $q_{1}/q_{2}=2^{13}$, so taking i=3 and α=1/5, we may once again apply this procedure to obtain $r=\left(21/40,\;19/40,\;\left(1+2^{-15}\right)/10,\;1/40+1/2^{14},\;1/2^{16},\;\cdots ,\;1/2^{16}\right)$ with $M\left(p\right)\geq M\left(q\right)\geq M\left(r\right)\geq 0$. Noting that now $r_{2}/r_{3}\geq 19/5=4.25$ and $r_{4}/r_{5}\geq 2^{16}/40=1638.4$, the procedure can continue, gradually trickling down mass from the head up to the tail.

We emphasize that the indices eligible for rebalancing change at every step and the indices that were once ineligible (such as 2 and 4) can become eligible at future steps. In fact, if we rebalance at Index 4, we notice that the fourth position in the newly created vector would be smaller than $(1+2^{-8})/50$, so Index 3 would once again become eligible. It follows that some indices can become eligible for rebalancing more than once.

In conclusion, in this paper, we strictly refined the work of [5] presenting a generalized approach in the form of Theorem 2, as well as a data-dependent numerical procedure resulting in tighter refinements. The rebalancing technique in Section 2.4 and Theorem 3 provides a significant improvement to the procedure in Theorem 2; thus, for future work, we propose a precise study of the convergence of this method on real data, as well as a follow-up work showing how the same method can be applied to other inequalities, such as Rioul’s inequality, Equation (4).
